# Plant Oxidative Stress: Biology, Physiology and Mitigation

**DOI:** 10.3390/plants11091185

**Published:** 2022-04-28

**Authors:** Mirza Hasanuzzaman, Masayuki Fujita

**Affiliations:** 1 Department of Agronomy, Faculty of Agriculture, Sher-e-Bangla Agricultural University, Dhaka 1207, Bangladesh; mhzsauag@yahoo.com; 2 Laboratory of Plant Stress Responses, Faculty of Agriculture, Kagawa University, Miki-cho 761-0795, Kita-gun, Kagawa, Japan

Due to climate change plants are frequently exposed to abiotic and biotic stresses, and these stresses pose serious threats to plant growth and productivity. A key sign of such stresses at the molecular level is the accelerated production of reactive oxygen species (ROS), which cause oxidative stress. Abiotic stresses result in the reduction of photosynthetic activities of the plants and accelerate the accumulation of ROS—which are oxygen radicals and their derivatives—such as hydrogen peroxide (H_2_O_2_) singlet oxygen (^1^O_2_), superoxide radicals (O_2_^•−^), and hydroxyl radical (OH^•^). These are highly reactive and usually toxic. The ROS can induce cellular injuries through protein oxidation, lipid peroxidation, and DNA damage, which finally may result in a plant’s cellular death [1,2,3]. Recently, ROS have emerged as major regulatory molecules in plants, and their role in early signaling events initiated by normal cellular metabolic function and environmental stress is now well established. Under normal circumstances, there is a balance between the generation and the elimination of ROS. However, this balance can be hampered by different biotic and abiotic stresses, resulting in the generation of a large number of ROS that should be counteracted by the antioxidant machinery in cells. Finding ways to enhance the antioxidant defense systems in plants is a very important task for plant biologists. Recent progress in plant molecular biology and biotechnology has been targeting the development of approaches that can be used to enhance the antioxidant defense systems in plants. New knowledge acquired through research on oxidative stress, abiotic stress, and biotic stress tolerance in plants will help us to apply stress-responsive determinants and to engineer plants with enhanced tolerance to stress.

Considering the deleterious effects of ROS, their detoxification is very important in order to ensure plant production. Enhanced activities of antioxidant enzymes play a crucial role in upregulating the adaptation mechanisms of plants under oxidative stress [4]. Non-enzymatic antioxidants—such as ascorbate (AsA), glutathione (GSH), tocopherols (Toc), flavonoids, and carotenoids—and enzymatic antioxidants—such as superoxide dismutase (SOD), monodehydroascorbate reductase (MDHAR), dehydroascorbate reductase (DHAR), ascorbate peroxidase (APX), catalase (CAT), glutathione reductase (GR), glutathione *S*-transferase (GST), glutathione peroxidase (GPX), and peroxidase (POD)—take part in scavenging ROS and protect the plants from oxidative damage [1,2].

Advanced molecular tools and genomics tools have shown that antioxidant defense is crucial for defending the plant against oxidative stress [5]. Many researchers have revealed that modern genomics studies have advanced our capabilities of improving crop genetics, especially those traits relevant to abiotic stress management. Billah et al. [5] reviewed the literature for updated and comprehensive studies concerning all possible combinations of advanced genomics tools and the gene regulatory network of reactive oxygen species homeostasis for the appropriate planning of future breeding programs, which will assist sustainable crop production under salinity and drought conditions [5].

From several studies, it is well established that the enhancement of antioxidant defense is highly correlated with stress tolerance. Seleiman et al. [6] reported that sequential application of proline (Pro), AsA, and/or GSH rectifies ion imbalance and strengthens antioxidant systems in salt-stressed cucumber by improving growth characteristics, photosynthetic efficiency, relative water content (RWC), and membrane stability index (MSI). Additional improvements were seen in AsA, Pro, and GSH; enzymatic activity; leaf and root K^+^ and Ca^2+^ contents; and their ratios to Na^+^, while the same sequential applications significantly reduced leaf and root Cd^2+^ and Na^+^ contents [6].

Some of extremophile and endemic plants were found to have ample capacity for antioxidant defense, and those plants provide enhanced tolerance to oxidative stress. Accumulation of secondary metabolites is a defense mechanism, as reported by Hashim et al. [7]. They found that endemic endangered species viz. *Nepeta septemcrenata*, *Origanum syriacum* subsp. *Sinaicum*, *Phlomis aurea*, *Rosa arabica*, and *Silene schimperiana*) showed elevated phenols, AsA, Pro, flavonoids, and tannins content in response to different altitudes. Secondary metabolites progressively increased in the studied species that were associated with a significant decrease in the levels of antioxidant enzyme activity [7].

ROS-induced oxidative damage can be alleviated by exogenous application of different chemical substances, such as amino acids and their derivatives, sugars, polyamines and vitamins, plant growth regulators to seeds (as seed priming), roots (as irrigation or soil incorporation), or leaves (as foliar application), at low concentrations. The systemic action of these chemicals improved various abiotic stress tolerances, including salinity, by increasing the antioxidant defense system and decreasing oxidative injuries at the cellular level [8].

Del Pino et al. [9] investigated the persistence of the effects of selenium (Se)-fertilization and found that Se biofortification increased the nutritional and qualitative values of foods in Se-deficient regions and increased the tolerance of oxidative stress in olive trees. This result indicated that trace elements have important functions in the adaptability of plants [9].

Selenium is a well-recognized trace element that has shown multifarious positive effects on plants. This element alone or in combination with other elements could provide increased plant oxidative stress tolerance [10]. The study conducted by Rahman et al. [11] revealed that supplementation of Se, boron (B), and Se + B enhanced the activities of APX, MDHAR, DHAR, GR, CAT, GPX, GST, POD, Gly I, and Gly II. This supplementation consequently diminished the H_2_O_2_ content and MDA content under salt stress and improved the growth parameters. The results reflected that exogenous Se, B, and Se + B enhanced the enzymatic activity of the antioxidant defense system and the glyoxalase systems under different levels of salt stress. This ultimately alleviated the salt-induced oxidative stress. Se+B supplementation was more effective than a single treatment [11].

Phytohormones are also important stress elicitors and there are many plant studies that show that exogenous application of phytohormones can mitigate oxidative stress in plants. Al-Harthi et al. [12] performed seed priming with gibberellic acid (GA) and jasmonic acid (JA) and, subsequently, the plants (summer squash) were grown in saline media. They observed that GA and JA resulted in a reduction of the concentration of Na^+^, Cl^−^, and the chlorophyll (Chl) *a*/*b* ratio. Increasing the activity of SOD, CAT, and APX; the quantities of K^+^ and Mg^2+^; the K^+^/Na^+^ ratio; and the quantities of RNA, DNA, Chl *b*, and carotene, ameliorated the growth of salinized plants [12].

Similarly, salicylic acid (SA) was also found to be very protective against oxidative stress due to its metabolic functions. This hormone was found to enhance antioxidant defense and osmolyte metabolism, which was the main cause of oxidative stress tolerance in watermelons exposed to B toxicity [13]. Exogenously applied SA promoted photosynthesis and, consequently, biomass production in watermelon seedlings treated with a high level of B by reducing B accumulation, lipid peroxidation, and the generation of H_2_O_2_, while significantly increasing levels of the most reactive ROS, OH^•^ [13].

The interactive effects of methyl jasmonate and SA were found to mitigate drought-induced oxidative damages in *Phaseolus vulgaris* as observed by Mohi-Ud-Din et al. [14]. Combined application of these phytohormones remarkably enhanced the drought tolerance of plants by improving the physiological activities and antioxidant defense system (SOD, CAT, POD, GPX, and GST as well as the enzymes of the AsA–GSH cycle). Phytohormones lowered the generation of O_2_^●−^ and H_2_O_2_ and the malondialdehyde (MDA) content [14].

Some of the non-enzymatic antioxidants, such as AsA, GSH, and Toc, were reported to be very effective in scavenging and detoxifying ROS. In okra, foliar spray of α-Toc showed an antioxidant potential, which promoted salt tolerance [15]. Foliar application of α-Toc significantly improved the yield in tested okra varieties by increasing the activity of antioxidants (CAT, GPX, SOD, and AsA), accumulation of glycine betaine, and total free Pro in fruit tissues under saline and non-saline conditions. However, these effects were dose-dependent [15].

Manipulating production practices or growing techniques can also help in mitigating oxidative stress in plants. Using a resistant cultivar or rootstock is one possible approach. Tao et al. [16] reported that the use of heat-resistant rootstock grafting is a viable technique that is practiced globally in order to improve plant resistance to abiotic stresses. Bitter melon (*Momordica charantia* L.)-grafted cucumber seedlings showed significantly improved heat-induced growth inhibition and photoinhibition, maintained better photosynthesis activity, and accumulated greater biomass than self-grafted seedlings [16].

Many amino acids and peptones are involved in the stimulation of physiological and metabolic functions of plants [17,18]. Peptone-induced cadmium (Cd) tolerance in *Spinacia oleracea* through modulation of antioxidant metabolism was reported by Emanuil et al. [18]. The application of peptone decreased Cd uptake and decreased levels of MDA, H_2_O_2_, and electrolyte leakage in spinach by increasing the activity of antioxidant enzymes. It indicated that peptone is a promising plant growth regulator that represents an efficient approach for the phytoremediation of Cd-polluted soils and enhancement of spinach growth, yield, and tolerance under a Cd-dominant environment [18].

Recently, nanoparticles (NPs) have shown increasing attention in research focusing on enhancing the growth progress and development of plants [19]. NPs have recently become more commonly employed in commercial products and industrial applications [20]. There are many studies where NPs are commonly used as growth regulators and stress elicitors [21]. In their study, Ahmad et al. [22] examined the effect of zinc oxide NPs in As-stressed soybean plants. Application of zinc oxide NPs to the As-stressed plants showed enhanced activities of the enzymes involved in the AsA–GSH cycle as well as SOD and CAT [22].

This Special Issue, “Plant Oxidative Stress: Biology, Physiology, and Mitigation” published 11 original research works and 1 review article that discussed the various aspects of ROS Biology, metabolism, and the physiological mechanisms and approaches to mitigating oxidative stress. These types of research studies show further directions for the development of crop plants that are tolerant to abiotic stress in the era of climate change.
